# Day–night variation and age-related differences in gadolinium-based contrast media enhancement in the brain: A T1 mapping study

**DOI:** 10.1371/journal.pone.0346730

**Published:** 2026-04-09

**Authors:** Dongjun Lee, Yangsean Choi, Eunseon Jeong, Yoonho Nam, Sungyang Jo, Sun Ju Chung, Ho-Sung Kim

**Affiliations:** 1 Department of Radiology and Research Institute of Radiology, University of Ulsan College of Medicine, Seoul, Republic of Korea; 2 Department of Biomedical Engineering, Hankuk University of Foreign Studies, Yongin-Si, Gyeonggi‐do, Republic of Korea; 3 Department of Neurology, Asan Medical Center, University of Ulsan College of Medicine, Seoul, Republic of Korea; Mae Fah Luang University School of Anti Aging and Regenerative Medicine, THAILAND

## Abstract

The glymphatic system clears metabolic waste from the brain and can be probed using contrast-enhanced MRI. We investigated whether early-phase gadolinium-based contrast agent (GBCA) uptake varies with time of day and age. In this retrospective study, 447 patients who underwent brain MRI for suspected movement disorders received pre- and post-contrast T1 mapping a median of 5.9 minutes after GBCA injection. Scans were categorized as daytime (n = 307) or nighttime (n = 140). Regional ΔT1 values were measured in the cerebral and cerebellar cortices, white matter, basal ganglia perivascular spaces, and choroid plexus. Daytime cohorts showed significantly greater cortical enhancement than nighttime cohorts, particularly in the frontal, parietal, and temporal lobes, while no differences were observed in white matter, perivascular spaces, or choroid plexus. Increasing age was independently associated with stronger cortical enhancement across both day and night scans, with no significant interaction between age and time. These findings suggest that cortical GBCA retention is influenced by both circadian timing and aging, supporting early-phase T1 mapping as a practical approach to evaluate human glymphatic function.

## Introduction

The glymphatic system is a brain-wide, glial-dependent fluid transport network responsible for clearing metabolic waste products, including amyloid-β and tau proteins, from the central nervous system [1–3]. It operates through periarterial influx, mixing with interstitial fluid (ISF), and perivenous clearance via meningeal lymphatic vessels, and depends critically on aquaporin-4 (AQP4) channels expressed on astrocytic endfeet [1,2,4–6]. Dysfunction of the glymphatic system has been associated with the accumulation of neurotoxic proteins and development of major neurodegenerative diseases, including Alzheimer’s and Parkinson’s diseases [1,7,8]. Consequently, investigating the mechanisms and dynamics of glymphatic clearance is increasingly important for understanding brain health and disease.

Gadolinium-based contrast agents (GBCAs) are routinely used in magnetic resonance imaging (MRI) to visualize tissue structures and pathologies. Although GBCAs do not typically cross an intact blood-brain barrier (BBB) [9], recent evidence demonstrates that they can rapidly penetrate the blood-cerebrospinal fluid (CSF) barrier at the choroid plexus (CP) within seconds to minutes after injection. From there, they enter perivascular spaces (PVS) and, driven by glymphatic flow, distribute into the brain parenchyma [10–12]. This early phase distribution of GBCA, observable within minutes of intravenous administration, likely reflects immediate CSF-ISF exchange dynamics and early glymphatic influx.

Previous studies have extensively applied GBCA imaging to evaluate glymphatic function. Initially, glymphatic imaging relied on invasive intrathecal administration of GBCAs, limiting clinical applicability [13–16]. However, recent advancements have enabled non-invasive glymphatic assessment using various intravenous contrast-enhanced MRI techniques, including T1 mapping, heavily T2-weighted FLAIR, and dynamic susceptibility contrast MRI. These approaches enabled visualization of GBCA entry into CSF compartments via the CP, subsequent distribution along PVS, and eventual interaction with the brain parenchyma [10,12,17,18]. Although earlier studies predominantly focused on delayed imaging phases (typically 3–24 h post-injection) to evaluate glymphatic clearance [17,18], recent evidence supports the value of early-phase imaging. Human and animal studies have demonstrated GBCA entry into the CSF within minutes of intravenous injection, most notably around the CP as early as 20s [12], followed by distribution into PVS [10,11]. These findings highlight the potential of early imaging to sensitively capture the initial phase of glymphatic transport, which may become less distinct as contrast redistributes over time.

Glymphatic activity is strongly influenced by sleep, with greater clearance during sleep than wakefulness [19]. In addition, glymphatic function may be modulated by intrinsic circadian rhythms, independent of sleep [20,21]. Wakefulness itself may transiently suppress glymphatic clearance, potentially resulting in greater early-phase parenchymal enhancement [17]. Therefore, it was hypothesized that daytime MRI scans would show greater early-phase GBCA enhancement (lower T1 values) than nighttime scans, reflecting reduced glymphatic clearance.

This study primarily aimed to quantitatively evaluate circadian variations in early-phase GBCA uptake in patients with suspected movement disorders using contrast-enhanced T1 mapping. Our secondary aims were to identify specific brain regions demonstrating distinct day–night differences in early-phase enhancement and investigate the influence of age on these circadian patterns, thereby providing insight into how aging may modulate diurnal patterns of brain fluid clearance.

## Materials and methods

### Study participants

**Fig 1:**
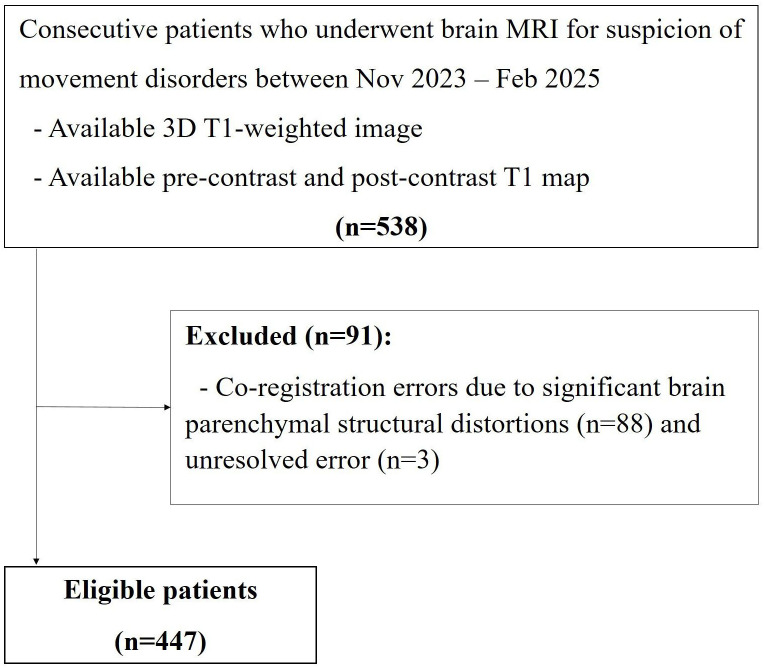
Flowchart of participant selection.

This single-center retrospective cohort study was approved by the Institutional Review Board of Asan Medical Center (IRB number: 2024−0465) and conducted in accordance with the Declaration of Helsinki and relevant guidelines and regulations. The data were accessed for research purposes on 19/03/2025. The authors did not have access to information that could identify individual participants during or after data collection. Owing to the retrospective design, the requirement for informed consent was waived. Consecutive participants who underwent brain MRI for clinical suspicion of movement disorders between November 2023 and February 2025 were retrospectively reviewed. The inclusion criteria were as follows: (1) availability of three-dimensional T1-weighted image and (2) pre-contrast and post-contrast T1 map. The exclusion criteria were as follows: 1) significant brain atrophy or encephalomalacia and 2) unresolved co-registration errors. Co-registration refers to the spatial alignment between pre- and post-contrast images, which is essential for accurate quantitative analysis. Co-registration errors primarily resulted from excessive patient motion during MRI acquisition, and cases where adequate alignment could not be achieved using the adopted registration method were excluded. Two experienced neurologists (blinded) diagnosed idiopathic Parkinson’s disease (IPD) according to the International Parkinson and Movement Disorder Society criteria [22], and essential tremor (ET) using the consensus statement criteria for tremor classification [23]. A flowchart of patient selection is illustrated in **Fig 1**.

### MRI acquisition

All MRI scans were performed on a 3T system equipped with a 64-channel head coil (Magnetom Vida, Siemens Healthineers, Erlangen, Germany). The imaging protocol comprised a pre-contrast Compressed Sensing Magnetization Prepared 2 Rapid Acquisition Gradient Echo (CS-MP2RAGE) sequence with multiplanar reconstructions of T1-weighted images and T1 mapping. A post-contrast CS-MP2RAGE was obtained a median of 5.9 minutes (interquartile range [IQR], 5.5–6.5) following intravenous injection of GBCA (Dotarem, Guerbet). All participants were instructed to remain awake and were unsedated during the MRI examination. Detailed imaging parameters are provided in **S1 Table**.

### Image preprocessing

Pre-contrast T1-weighted images were registered to post-contrast T1-weighted images using a rigid-body transformation approach with 6° of freedom, implemented via FMRIB’s Linear Image Registration Tool and optimized using a least-squares cost function [24–26]. The resulting transformation matrix was applied to both pre-contrast T1-weighted images and T1 maps to ensure spatial alignment with post-contrast images. Following registration, cortical and white matter segmentations were performed on the registered pre-contrast T1-weighted images using FreeSurfer’s SynthSeg [27], a deep learning-based segmentation tool for parcellating brain structures for subsequent quantitative analysis of contrast enhancement.

### Deep learning-based segmentation method

A deep learning-based model was developed for automated segmentation of the CP and basal ganglia PVS. For model training, ground truth labels were generated in 101 patients, and the trained model was applied to segment CP and basal ganglia PVS across the entire dataset. In brief, initial CP and lateral ventricle masks were generated using FreeSurfer [28], which were refined using a Gaussian Mixture Model-based approach [29]. Basal ganglia PVS were segmented using a multi-step algorithm. First, T1-weighted images were standardized using histogram equalization. A Frangi filter [30] was then applied to detect tubular structures characteristic of PVS, using scale parameters optimized for basal ganglia PVS (scale range: 1–2). Basal ganglia masks were further refined through morphological operations (binary closing followed by dilation with a spherical structuring element) to ensure comprehensive coverage. A threshold was defined as the mean vesselness response within these masks plus one standard deviation. Voxels exceeding this threshold were classified as basal ganglia PVS, with separate labels assigned to the left and right hemispheres. Final CP and basal ganglia PVS masks were reviewed and corrected by an expert radiologist (blinded) to ensure anatomical accuracy and proper delineation of these structures.

### Deep learning model training

**Fig 2:**
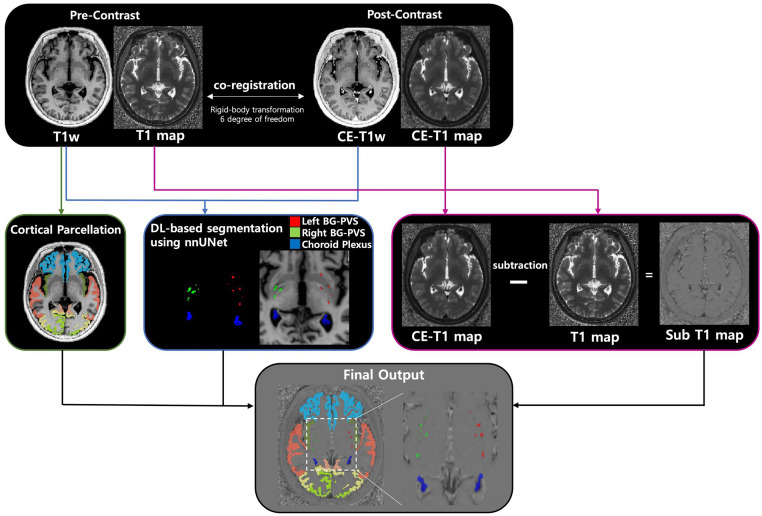
Schematic workflow of image preprocessing.

The manually refined CP and basal ganglia PVS masks were used to train an nnU-Net model for automated segmentation [31]. Training employed dual-channel inputs, consisting of co-registered pre-contrast and post-contrast T1-weighted images. The training dataset comprised 101 paired images, and hyperparameters were optimized using five-fold cross-validation. The finalized model was then applied to the full dataset for inference. A blinded expert radiologist independently reviewed each segmentation output, and corrected false-positive and false-negative results. The overall workflow is illustrated in **Fig 2**.

### Statistical analysis

Data are presented as mean with standard deviation, median with interquartile range, or counts with percentages, as appropriate. Baseline characteristics between daytime and nighttime cohorts were compared using the Mann–Whitney U test for continuous variables and chi-square or Fisher’s exact test for categorical variables. The daytime and nighttime cohorts were defined based on our institution’s clinical MRI operational shifts (daytime: 06:00–17:59; nighttime: 18:00–05:59), which broadly correspond to typical societal activity–rest periods.

Regional differences in early-phase GBCA enhancement (ΔT1) between daytime and nighttime scans were evaluated using analysis of covariance, with age, sex, and clinical diagnosis included as covariates. Covariates were selected a priori: age (a known determinant of glymphatic function [4,32]), sex (potential sex-related differences in brain fluid dynamics [33]), and clinical diagnosis (to account for the heterogeneous diagnostic composition and significant between-group difference in diagnostic distribution). The effect of age on cortical ΔT1 values was examined using linear regression analyses performed separately for the daytime and nighttime cohorts, adjusting for sex and clinical diagnosis. Regression coefficients (β) were reported as the change in ΔT1 per one-year increase in age. To determine whether age-related slopes differed between groups, interaction analyses were performed by including an age × scan time term in the models.

All tests were two-sided, and statistical significance was defined as *P* < 0.05. All statistical analyses were performed using R software (version 4.3.2; R Foundation for Statistical Computing, Vienna, Austria).

## Results

### Characteristics of the study participants

Overall, 447 participants were included in the final analysis. The mean age was 63.0 ± 13.3 years, and 199 participants were male (44.5%) and 248 were female (55.5%). Clinical diagnoses included IPD (n = 148, 33.1%), ET (n = 81, 18.1%), multiple system atrophy (n = 52, 11.6%), progressive supranuclear palsy (n = 13, 2.9%), and vascular parkinsonism (n = 10, 2.2%). The remaining 143 participants (32.0%) were classified under other diagnoses.

With respect to MRI acquisition timing, daytime scans accounted for 307 cases (68.7%), whereas nighttime scans accounted for 140 cases (31.3%). Detailed baseline characteristics of the participants are presented in **Table 1**.

**Table 1:** Baseline clinical characteristics of the study participants.

Variables	n = 447
Age	63.0 ± 13.3
Sex, n (%)	
Male	199 (44.5)
Female	248 (55.5)
Diagnoses, n (%)	
Idiopathic Parkinson’s disease	148 (33.1)
Essential tremor	81 (18.1)
Multiple system atrophy	52 (11.6)
Progressive supranuclear palsy	13 (2.9)
Vascular parkinsonism	10 (2.2)
Others	143 (32.0)
MRI acquisition period*, n (%)	
Daytime	307 (68.7)
Nighttime	140 (31.3)

MRI, magnetic resonance imaging.

* Daytime was defined as 06:00–17:59, and nighttime as 18:00–05:59.

### Baseline comparison of daytime and nighttime cohorts

Baseline characteristics stratified by MRI acquisition time are presented in **Table 2**. The daytime (n = 307) and nighttime (n = 140) cohorts were comparable, showing no significant differences in age (*P* = 0.853) or sex distribution (*P* = 0.562). However, a significant difference was observed in the distribution of clinical diagnoses (*P* = 0.007). Post-hoc analysis indicated that this difference was primarily driven by the lower proportion of patients with ET in the nighttime group (21.0%) compared with that of patients with IPD (43.2%; Bonferroni-adjusted *P* = 0.013). Therefore, this variable was included as a covariate in subsequent analyses to control for its potential influence. Post-contrast acquisition timing was comparable between groups, with a median delay of 5.9 minutes (IQR, 5.5–6.5) during daytime and 6.0 minutes (IQR, 5.5–6.4) during nighttime (*P* = 0.660).

**Table 2:** Clinical variables by MRI acquisition time.

Variables	Day (n = 307)	Night (n = 140)	*P*-value
Age, median [IQR]	65.0 [59.0; 72.0]	66.0 [57.5; 72.0]	0.853
Sex, n (%)			0.562
Male	140 (45.6)	59 (42.1)	
Female	167 (54.4)	81 (57.9)	
Diagnoses, n (%)			0.007
Idiopathic Parkinson’s disease	84 (27.4)	64 (45.7)	
Essential tremor	64 (20.8)	17 (12.1)	
Multiple system atrophy	39 (12.7)	13 (9.3)	
Progressive supranuclear palsy	10 (3.3)	3 (2.1)	
Vascular parkinsonism	7 (2.3)	3 (2.1)	
Others	103 (33.6)	40 (28.6)	

MRI, magnetic resonance imaging; IQR, interquartile range.

*The relative proportion of patients diagnosed with idiopathic Parkinson’s disease and essential tremor differed significantly between the day and night groups (pairwise Fisher’s exact test, Bonferroni-adjusted *P* = 0.013).

### Day-night differences in early-phase GBCA enhancement

The primary outcome of the study was the difference in early-phase GBCA enhancement (ΔT_1_) between daytime and nighttime cohorts, with results summarized in **Table 3**. After adjusting for age, sex, and clinical diagnoses, participants scanned during the day exhibited significantly greater GBCA enhancement in the cortex compared with those scanned at night. This effect was consistently observed in global measures of the cerebral cortex (adjusted *P* = 0.022) and cerebellar cortex (adjusted *P* = 0.042). Regional analysis further localized this pattern to the frontal (adjusted *P* = 0.031), parietal (adjusted *P* = 0.007), and temporal (adjusted *P* = 0.041) cortical lobes. In contrast, no day-night difference was observed in other brain structures. No significant differences in enhancement were found in the cerebral white matter (adjusted *P* = 0.726), cerebellar white matter (adjusted *P* = 0.594), basal ganglia PVS (adjusted *P* = 0.860), or CP (*P* = 0.672). Unadjusted comparisons yielded broadly similar findings.

**Table 3:** Regional ΔT_1_ values compared by MRI acquisition time.

Brain region (median [IQR])	Day (n = 307)	Night (n = 140)	Unadjusted *P*-value	Adjusted *P*-value
**Global cortical measures**				
Cerebral cortex	−166.4 [−180.9;-151.7]	−160.9 [−175.1;-150.6]	0.031	0.022
Cerebellar cortex	−174.6 [−189.7;-156.8]	−165.0 [−184.5;-153.3]	0.029	0.042
**Regional cortical lobes**				
Frontal cortex	−147.5 [−164.5;-131.1]	−144.0 [−157.1;-125.9]	0.048	0.031
Parietal cortex	−169.0 [−182.7;-152.4]	−163.3 [−176.9;-147.0]	0.013	0.007
Temporal cortex	−170.2 [−184.7;-156.9]	−166.7 [−179.9;-154.3]	0.102	0.041
Occipital cortex	−229.3 [−251.7;-207.8]	−223.1 [−250.0;-204.3]	0.234	0.221
**Cerebral and cerebellar white matter**				
Cerebral white matter	−39.3 [−44.6;-32.6]	−38.4 [−44.9;-33.4]	0.754	0.726
Cerebellar white matter	−50.4 [−59.2;-44.7]	−50.3 [−57.6;-43.6]	0.602	0.594
**Basal ganglia perivascular spaces**	−87.2 [−102.5;-75.2]	−88.5 [−103.4;-74.8]	0.861	0.860
**Choroid plexus**	−869.7 [−937.7;-791.9]	−869.2 [−937.1;-812.2]	0.646	0.672

MRI, magnetic resonance imaging; IQR, interquartile range; ANCOVA, analysis of covariance.

*Adjusted *P*-values were calculated using ANCOVA after adjustment for age, sex, and clinical diagnoses.

### Association Between age and cortical GBCA enhancement by scan time

**Fig 3:**
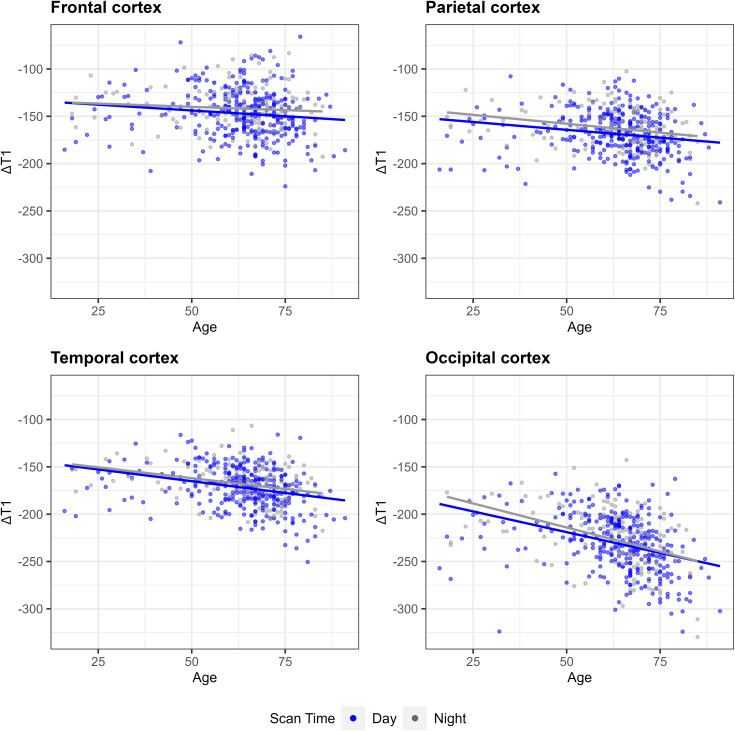
Scatterplots of ΔT_1_ versus age in the cerebral cortex. Scatterplots depict the relationship between age (x-axis) and change in T1 relaxation time (ΔT1, y-axis) across four distinct cortical regions. In each plot, data points and their corresponding linear regression lines are stratified by scan time: daytime (blue) and nighttime (gray). Across all regions, a consistent negative correlation indicates that GBCA enhancement becomes more pronounced with increasing age, a trend irrespective of scan time.

To investigate the influence of age on early-phase GBCA enhancement, linear regression analyses were performed stratified by scan time, evaluating the association between age and regional cortical ΔT1 values while adjusting for sex and clinical diagnosis. Detailed results for analyzed regions are presented in **Table 4**. Associations in the four major cortical regions are visualized in **Fig 3**.

**Table 4:** Linear regression analysis of age and regional ΔT1 values by scan time.

Brain region	Day (n = 307)		Night (n = 140)		Interaction P-value
	β-coefficient	*P*-value	β-coefficient	*P*-value	
**Global cortical measures**					
Cerebral cortex	−0.47	< 0.001	−0.34	0.014	0.533
Cerebellar cortex	0.02	0.891	0.09	0.567	0.692
**Regional cortical lobes**					
Frontal cortex	−0.34	0.004	−0.04	0.784	0.337
Parietal cortex	−0.39	< 0.001	−0.44	0.003	0.986
Temporal cortex	−0.55	< 0.001	−0.37	0.004	0.356
Occipital cortex	−0.94	< 0.001	−1.09	< 0.001	0.652
**Cerebral and cerebellar white matter**					
Cerebral white matter	−0.15	< 0.001	−0.16	0.017	0.66
Cerebellar white matter	−0.16	0.001	−0.11	0.205	0.657
**Basal ganglia perivascular spaces**	−0.51	< 0.001	−0.29	0.07	0.286
**Choroid plexus**	−0.90	0.079	−1.66	0.014	0.601

Adjusted for sex and clinical diagnosis.

During daytime scans, age was significantly associated with lower ΔT1 values, indicating greater GBCA enhancement, across all analyzed cortical regions: frontal (β = –0.34, *P* = 0.004), parietal (β = –0.39, *P* < 0.001), temporal (β = –0.55, *P* < 0.001), and occipital cortices (β = –0.94, *P* < 0.001).

During nighttime scans, significant associations persisted in the parietal (β = –0.44, *P* = 0.003), temporal (β = –0.37, *P =* 0.004), and occipital cortices (β = –1.09, *P* < 0.001), whereas no significant relationship was observed in the frontal cortex (β = –0.04, *P* = 0.784).

Despite these regional differences, interaction analyses revealed no significant differences in the age-related slopes between daytime and nighttime cohorts (all interaction *P* > 0.05), indicating that the effect of aging on early-phase GBCA enhancement was consistent regardless of scan time.

## Discussion

This study provides quantitative evidence that very early-phase parenchymal enhancement of intravenously administered GBCA is influenced by time of day. GBCA enhancement, reflected by greater reductions in T1 relaxation time (ΔT1), was significantly more pronounced in the cerebral and cerebellar cortices of individuals scanned during daytime compared with that of nighttime. In addition, advancing age was consistently associated with stronger enhancement across most cortical regions. Collectively, these findings are consistent with the hypothesis that glymphatic function may exhibit diurnal variations and progressively decline with aging, though the modest effect sizes and retrospective design warrant cautious interpretation.

The present findings are consistent with intrinsic circadian modulation beyond the immediate arousal state [20]. As participants were imaged while awake, the observed day-night differences are unlikely to be explained solely by concurrent sleep during acquisition. This interpretation converges with recent findings from a non-contrast MRI approach: Ko et al. using DTI-ALPS demonstrated lower daytime ALPS indices, a proxy for perivascular water diffusivity, compared with nighttime [21]. The concordance of results from two distinct methodologies strengthens the argument that diurnal regulation of brain fluid dynamics may reflect a genuine biological phenomenon rather than a method-specific effect.

The methodological approach in this study differs from prior delayed-phase studies that focus on net clearance. For instance, a prospective study by Lee et al. in healthy volunteers, using imaging at 2 and 12 h post-injection, showed that a night of sleep enhances net GBCA elimination [17]. In contrast, the present study examined a single early time point to assess initial tracer dynamics. This emphasis on early-phase enhancement is supported by animal research from Taoka et al., who demonstrated in rats that parenchymal enhancement from intravenous GBCA peaks significantly later than the initial influx into the CSF, indicating a distinct early phase of contrast distribution [34]. The biological validity of capturing such early dynamics in humans is reinforced by findings from Sun et al., which demonstrated GBCA entry into the CSF within seconds to minutes of injection [12]. By focusing on the initial distribution and short-term retention of the contrast agent, our design is well-suited to detect the immediate suppression of clearance that occurs during the day, and it remains complementary to studies that quantify net elimination over longer intervals.

A plausible biological mechanism for this diurnal modulation is the circadian regulation of noradrenergic tone from the locus coeruleus [20,35]. This control by the brain’s central clock results in noradrenergic signaling peaking during the biological day to promote active wakefulness. Elevated norepinephrine is known to diminish the effective interstitial volume via astrocytic mechanisms, thereby increasing hydraulic resistance and attenuating convective fluid exchange [19,36]. The anatomical specificity of the findings strongly supports this interpretation, as significant day-night differences were confined to the cerebral and cerebellar gray matter, territories with prominent perivascular astroglial interfaces characterized by AQP4-polarized endfeet that are critical for this process [37]. Within this framework, higher daytime noradrenergic tone would favor a relative compaction of the ISF in AQP4-rich areas, impeding early clearance of GBCA from the parenchyma and manifesting as greater short latency retention.

No day-night difference was detected in CP enhancement levels (adjusted *P* = 0.672), which serves as an internal control for initial tracer entry into the CSF. This finding suggests that early influx is comparable between daytime and nighttime cohorts, and that downstream clearance dynamics within the parenchyma likely drive the cortical effects. Notably, although the age slope for CP reached significance within the nighttime cohort, the age × scan-time interaction was not significant (*P* = 0.601; Table 4), indicating that the age–ΔT1 relationship did not differ by scan time. Thus, aging consistently increases early retention, as reflected by greater enhancement, whereas the relative magnitude of diurnal fluctuation appears preserved. These observations imply that chronic age-related deterioration and acute circadian modulation may be governed by partially distinct mechanisms.

This study has several limitations. First, the retrospective, single-center design and inclusion of patients with suspected movement disorders may limit generalizability. Although known confounders were adjusted for, the inclusion of patients with various movement disorders and differing diagnostic distributions between groups could introduce unmeasured biases. Second, clock time was used as a proxy for circadian phase, which does not account for individual variations in sleep patterns or the potential influence of medications on glymphatic activity. Additionally, although all participants were instructed to remain awake and unsedated, wakefulness could not be objectively verified during the scan, and brief episodes of drowsiness or microsleep cannot be excluded. Third, the use of a single early post-contrast time point provides only a snapshot of GBCA retention and does not permit a full influx-efflux kinetic analysis. Fourth, the ΔT1 measurement reflects a composite of intravascular, perivascular, and interstitial compartments, preventing complete separation of glymphatic clearance from subtle, age-related changes in BBB permeability. Fifth, circadian variations in cerebral blood flow, blood pressure, body temperature, and hydration status may have influenced GBCA delivery and tissue enhancement independently of glymphatic function [38,39], though the absence of day–night differences in choroid plexus enhancement suggests that systemic GBCA delivery was comparable between groups. Finally, our findings were not validated in an independent cohort, and external validation is warranted.

In conclusion, early-phase cortical GBCA enhancement appears to be modestly greater during the day than at night and increases with age. This pattern is consistent with a clearance-limited interpretation, in which aging may be associated with chronically impaired clearance, and nocturnal states may acutely facilitate clearance. Prospective longitudinal studies incorporating objective sleep and circadian measures, together with time-resolved imaging, are warranted to further delineate these mechanisms and their clinical implications.

## Supporting information

S1 Table MRI acquisition parameters. (DOCX)
