# Supplementary material for: Day–night variation and age-related differences in gadolinium-based contrast media enhancement in the brain: A T1 mapping study
Source: PLoS One. 2026 Apr 9;21(4):e0346730. doi: 10.1371/journal.pone.0346730 (PMC13065030; doi:10.1371/journal.pone.0346730)
Supplement: S1 Table — (DOCX) [file pone.0346730.s001.docx]

**S1 Table. MRI acquisition parameters**

|  | 3D CS-MP2RAGE | 3D FLAIR | 2D T2 TSE | SWI |
| --- | --- | --- | --- | --- |
| Orientation | Sagittal | Sagittal | Axial | Axial |
| FOV (mm) | 256 × 240 | 256 × 216 | 210 × 210 | 256 × 176 |
| TR (ms) | 5000 | 5000 | 5910 | 40.7 |
| TE (ms) | 2.9 | 328 | 100 | 5.42–36.80 |
| TI (ms) | TI1 / TI2 700 / 2500 | 1600 |  |  |
| FA (degree) | FA1 / FA2 4 / 5 | FA1 / FA2 4 / 5 | 150 | 20 |
| Slice thickness (mm) | 1 | 1 | 4 | 2 |
| Matrix size | 256 × 240 | 256 × 216 | 512 × 358 | 480 × 480 |
| Scan time (min : sec) | 3:30 | 3:29 | 2:17 | 4:50 |

CS-MP2RAGE, Compressed Sense-Magnetization Prepared 2 Rapid Acquisition Gradient Echoes; SWI, susceptibility-weighted image; FOV, field of view; TR, repetition time; TE, echo time; TI, inversion time; FA, flip angle
